# Canine dorsal root ganglia satellite glial cells represent an exceptional cell population with astrocytic and oligodendrocytic properties

**DOI:** 10.1038/s41598-017-14246-7

**Published:** 2017-10-24

**Authors:** W. Tongtako, A. Lehmbecker, Y. Wang, K. Hahn, W. Baumgärtner, I. Gerhauser

**Affiliations:** 1 Department of Pathology, University of Veterinary Medicine Hannover, Bünteweg 17, D-30559 Hannover, Germany; 2 Center of Systems Neuroscience Hannover, Hannover, Germany

## Abstract

Dogs can be used as a translational animal model to close the gap between basic discoveries in rodents and clinical trials in humans. The present study compared the species-specific properties of satellite glial cells (SGCs) of canine and murine dorsal root ganglia (DRG) *in situ* and *in vitro* using light microscopy, electron microscopy, and immunostainings. The *in situ* expression of CNPase, GFAP, and glutamine synthetase (GS) has also been investigated in simian SGCs. *In situ*, most canine SGCs (>80%) expressed the neural progenitor cell markers nestin and Sox2. CNPase and GFAP were found in most canine and simian but not murine SGCs. GS was detected in 94% of simian and 71% of murine SGCs, whereas only 44% of canine SGCs expressed GS. *In vitro*, most canine (>84%) and murine (>96%) SGCs expressed CNPase, whereas GFAP expression was differentially affected by culture conditions and varied between 10% and 40%. However, GFAP expression was induced by bone morphogenetic protein 4 in SGCs of both species. Interestingly, canine SGCs also stimulated neurite formation of DRG neurons. These findings indicate that SGCs represent an exceptional, intermediate glial cell population with phenotypical characteristics of oligodendrocytes and astrocytes and might possess intrinsic regenerative capabilities *in vivo*.

## Introduction

Since the discovery of glial cells over a century ago, substantial progress has been made in understanding the origin, development, and function of the different types of glial cells in the central nervous system (CNS) and peripheral nervous system (PNS)^1^. Similar to neurons, astrocytes and oligodendrocytes are of neuroectodermal origin^2^, whereas peripheral Schwann cells, olfactory ensheathing cells (OECs), and satellite glial cells (SGCs) arise from the neural crest^3,4^. Microglial cells are derived from mesenchymal precursors of the yolk sac, which invade the nervous system in the fetal period^5,6^. In addition, the CNS contains Schwann cell-like glia (Synonyms: Aldynoglia, Schwann cell-like brain glia, central nervous system Schwann cells) that emerge in response to axonal damage in demyelinating diseases^7–9^. Initially regarded as non-excitable cells scaffolding and feeding neurons, glial cells have turned out to actively participate in brain function modulating neuronal communication by multiple mechanisms such as the production of glial neurotransmitters^10^. In addition, glial cells play an important role in the pathogenesis of various diseases and disorders of the human CNS including Alzheimer disease, multiple sclerosis, stroke, epilepsy, and spinal cord injury, which cause severe and often progressive disabilities in millions of patients worldwide^11–14^. Similarly, glial cells are involved in the immune pathogenesis of several idiopathic, infectious, and traumatic canine CNS diseases^15,16^.

The increasing knowledge of glial cell capacities in CNS homeostasis and disease prompted the idea of using peripheral glial cells including Schwann cells and OECs in transplantation-based therapies for spinal cord trauma^4,17^. However, therapeutic success compared to promising preclinical data based on studies in rodent models remains limited^18–21^. The reasons for this frequent observation are generally unknown and might be related to morphological and physiological differences between the rodent and human CNS limiting data extrapolation. In contrast, structure and organization of the canine and human CNS is similar to a large extent and recent studies demonstrated that species-specific properties of human glia are closer related to dogs than rodents^21–23^. In addition, some human CNS diseases including spontaneous spinal cord injury and multiple sclerosis have spontaneously occurring counterparts in dogs with comparable pathogenic mechanisms, lesion appearance, and clinical signs^16,24–26^. Consequently, the dog represents a valuable translational large animal model to study the pathogenesis of certain human inflammatory and degenerative CNS diseases and bridge the gap to rodent models^16,21,25,27,28^.

The dorsal roots of the spinal cord contain sensory ganglia, which are composed of afferent neurons, ensheathing SGCs, and connective tissue cells^29^. These dorsal root ganglia (DRG) neurons and SGCs form a unique structural unit^30^, representing the basis for their intense bidirectional communication^31^. Similar to astrocytes in the CNS, SGCs control the microenvironment of DRG neurons and functionally substitute the lacking blood-brain barrier in sensory ganglia^29^. Moreover, they can form perikaryal myelin sheaths and even possess phagocytic activity, which are typical functions of oligodendrocytes and microglia, respectively^32–34^. The aims of the present study were to characterize canine SGCs *in situ* and *in vitro*, and to compare these results to murine, and as far as available to simian SGCs and to investigate the potential role that SGCs might play in regenerative medicine as possible cell transplantation candidates.

## Results

### *In situ* characterization of SGCs

#### Dogs

**Figure 1:**
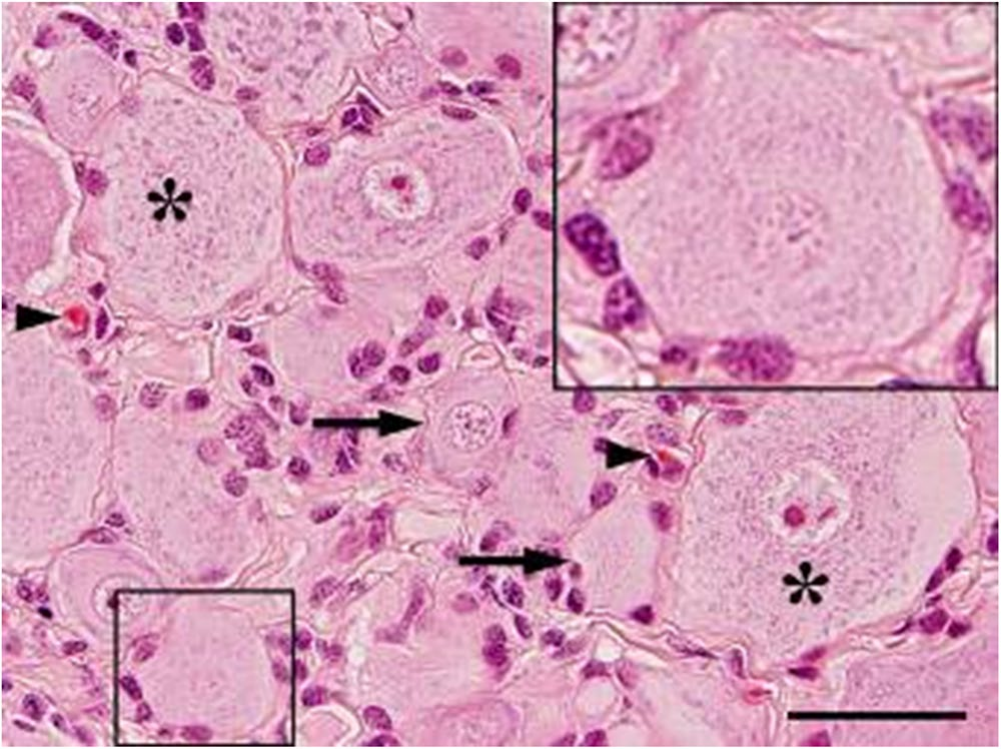
Dorsal root ganglion of a Beagle dog. Multiple large (>40 μm; asterisks) and small neurons (<40 μm, arrows) surrounded by a satellite glial cell sheath (insert). Note few fibroblasts and capillaries (arrowheads) in the interstitial stroma. Hematoxylin and eosin staining. Bar, 40 μm.

**Figure 2:**
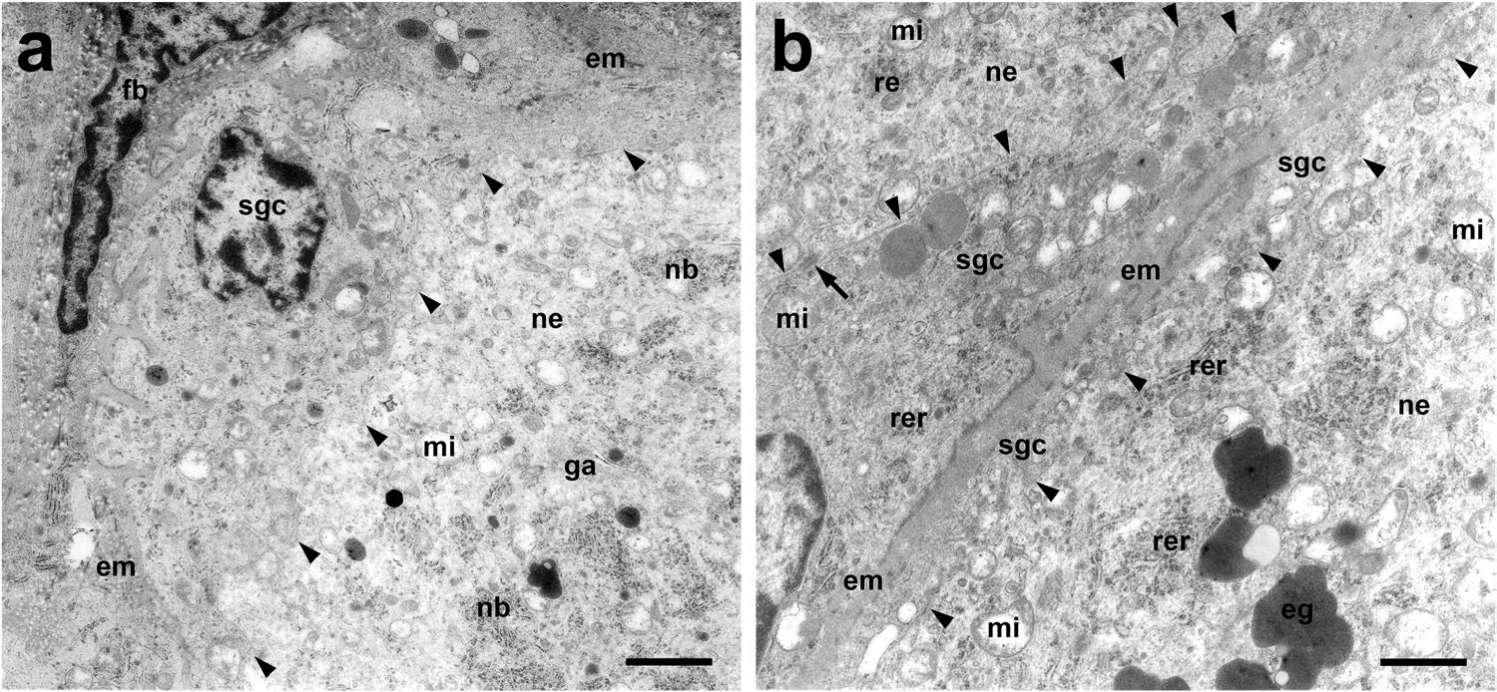
Dorsal root ganglion of an adult Beagle dog. Transmission electron microscopy. (**a)** Large neuron with adjacent SGC and fibroblast within connective tissue. Note the closely-spaced cytoplasmic membranes of neuron and SGC (arrowheads). Bar, 2 μm. (**b)** Two large neurons with SGC sheaths demarcated by connective tissue. Note the closely-spaced interdigitating cytoplasmic membranes (arrowheads) linked by desmosomes (arrow). Bar, 1 μm. eg, electron-dense granule; em, extracellular matrix; fb, fibroblast; ga, golgi apparatus; mi, mitochondrium; nb, Nissl body; ne, neuron; rer, rough endoplasmic reticulum; sgc, satellite glial cell.

Neurons were completely ensheathed by a rim of SGCs (Fig. 1). At the ultrastructural level, the interdigitating cytoplasmic membranes of neurons and SGCs were arranged in close juxtaposition and linked by multiple desmosomes (Fig. 2). This structural unit was surrounded by connective tissue composed of fibroblasts, blood vessels, and an extracellular matrix with abundant collagen fibers, which also contained large axons enwrapped by thick myelin sheaths. The electron-lucent cytoplasm of small and large neurons contained normal cellular organelles (nucleus, Golgi apparatus, smooth endoplasmic reticulum (ER), rough ER arranged in multiple Nissl bodies, mitochondria) and different numbers/densities of electron-dense granules (Fig. 2).

**Figure 3:**
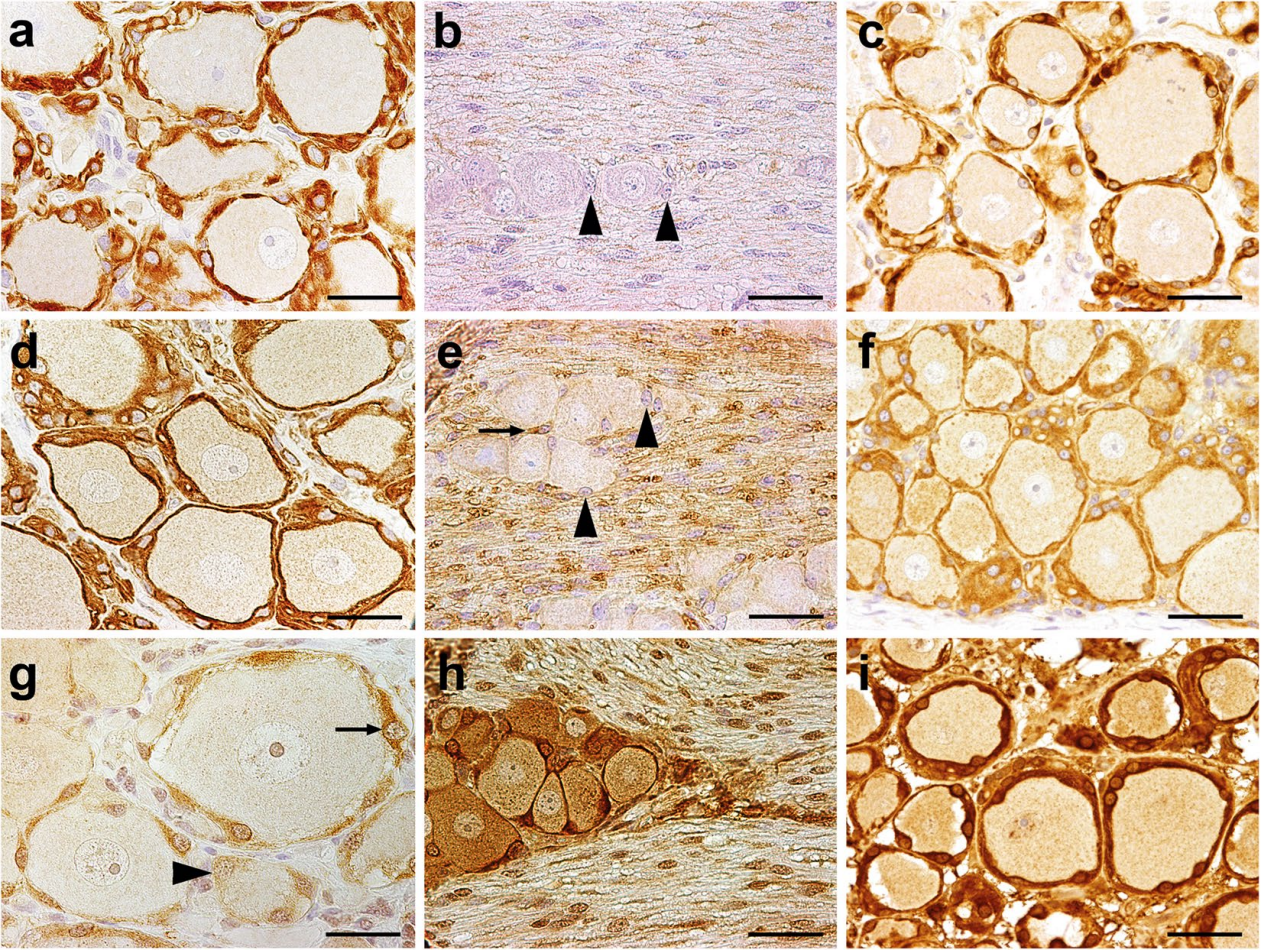
Dorsal root ganglion of a Beagle dog (**a**,**d**,**g**), a C57BL/6 mouse (**b**,**e**,**h**), and a gray langur (*Semnopithecus sp*.) (**c**,**f**,**i**). (**a**–**c**) Glial fibrillary acidic protein (GFAP), 2′,3′-cyclic-nucleotide 3′-phosphodiesterase (CNPase) (**d**–**f**), and (**g**–**i**) glutamine synthetase (GS). Most satellite glial cells (SGCs) are immunopositive for GFAP and CNPase in dogs and monkeys, whereas most murine SGCs lack expression of these markers (arrowheads). Note single CNPase^+^ SGC (arrow, **e**). In contrast, GS represents a good marker for murine and simian SGCs but not for canine SGCs. There are SGCs positive (arrow) and negative for GS (arrowhead) in dogs. Immunohistochemistry using the avidin-biotin-peroxidase complex method, the chromogen 3,3′-diamino-benzidine, and Mayer’s hematoxylin as counterstain. Bars, 40 μm.

**Figure 4:**
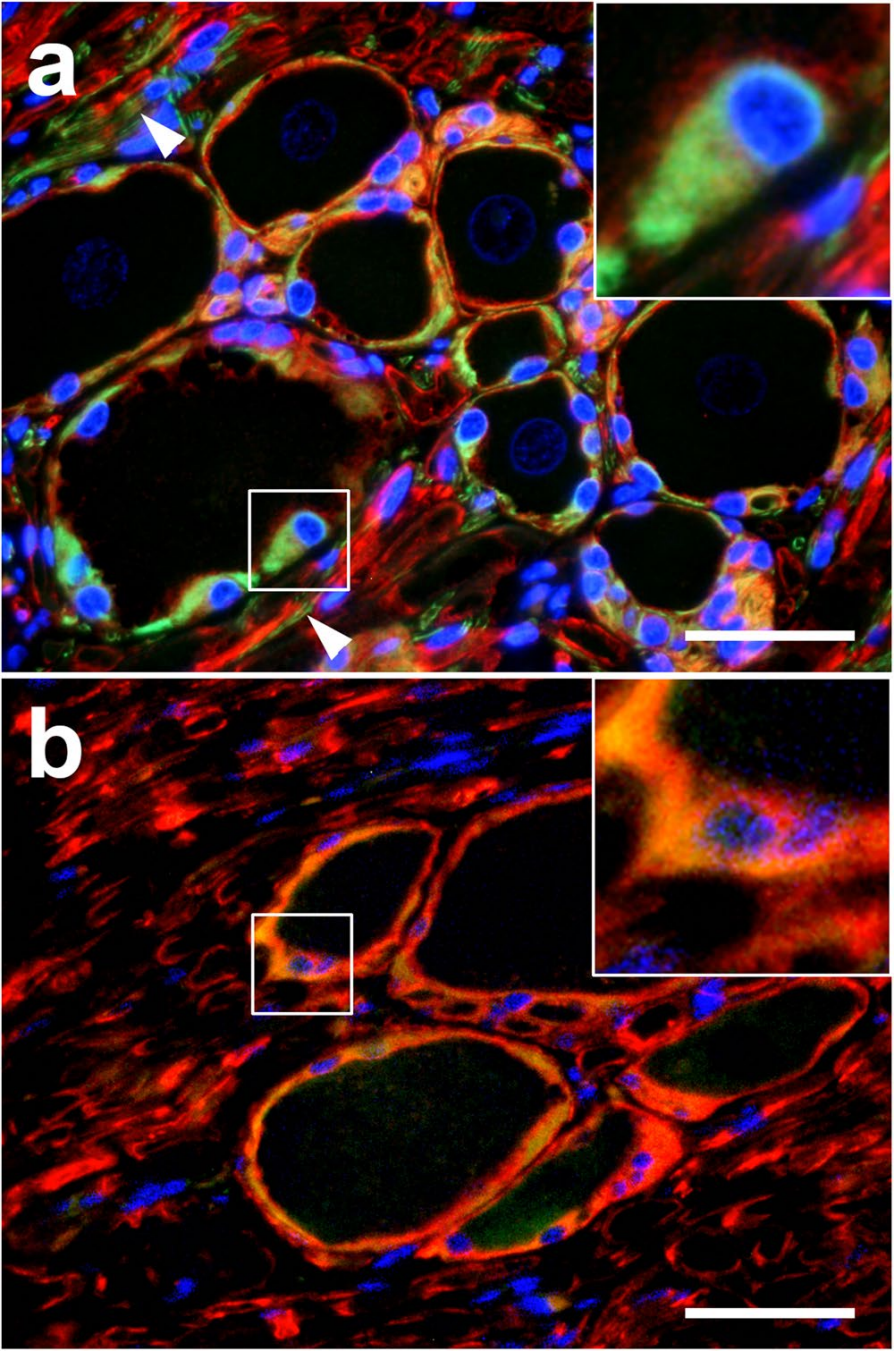
Dorsal root ganglion of a Beagle dog. (**a)** 2′,3′-cyclic-nucleotide 3′-phosphodiesterase (CNPase; red) and glial fibrillary acidic protein (GFAP; green) double-staining. Most satellite glial cells (SGCs) have a strong perinuclear CNPase and cytoplasmic GFAP expression (insert). Note CNPase^+^ nerve fibers surrounded by GFAP^+^ cells (arrowheads). **(b)** CNPase (red) and Nestin (green) double-staining. Most SGCs show a strong co-expression of these markers indicated by yellow staining (insert). Immunofluorescence double-labelling of the dorsal root ganglion of a beagle dog *in situ* with bisbenzimide as nuclear counterstain. Bar, 40 μm.

SGCs were mostly immunopositive for vimentin (median 85%; range: 84–88%; see Supplementary Fig. S2a), GFAP (78%; 73–89%; Fig. 3a), CNPase (93%; 86–97%; Fig. 3d), and Sox2 (83%; 80–91%; see Supplementary Fig. S2d). 44% (25–52%) and 11% (3–38%) of the SGCs expressed glutamine synthetase (GS; Fig. 3g) and S-100 protein (see Supplementary Fig. S2c), respectively. A high percentage of SGCs expressed interferon stimulated gene 15 (ISG15; 76%; 73–79%) and signal transducer and activator of transcription 1 (STAT1; 72%; 70–74%) in the nucleus as well as 2′-5′ oligoadenylate synthetase 1 (OAS1; 83%; 81–96%), protein kinase R (PKR; 77%; 72–80%), and STAT2 (10%; 10–11%) in the cytoplasm. In addition, the antiviral Mx protein was found in the cytoplasm of canine SGCs (28%; 21–31%). Few cells within the DRG reacted positive with antibodies directed against periaxin (5%; 4–8%), p75^NTR^ (1%; 0–3%), ionized calcium-binding adapter molecule 1 (Iba-1; 5%; 3–7%), and CD3 (3%; 0–4%). Major histocompatibility complex (MHC) class II proteins were also found in a small number of canine SGCs (18%; 17–21%). No immunoreaction was detected for human natural killer-1 (HNK-1; CD57) and the B cell markers CD79α and paired box 5 (Pax5) in SGCs. Immunofluorescence revealed a co-expression of CNPase and GFAP (Fig. 4a) and also of CNPase and Nestin (Fig. 4b) in the majority of canine SGCs.

#### Mice and monkeys

Similar to dogs, murine and simian SGCs were forming a glial cell sheath surrounding neurons (see Supplementary Fig. S3). A high number of murine SGCs expressed GS *in situ* (71%; 70–72%; Fig. 3h), whereas these cells show a low expression of CNPase (5%; 4–6%; Fig. 3e) and no expression of GFAP (Fig. 3b). In contrast, the majority of simian SGCs express GS (94%; 90–98%; Fig. 3i), CNPase (92%; 85–94%; Fig. 3f), and GFAP (80%; 78–84%; Fig. 3c). In addition, vimentin can be found in most simian SGCs (88%; 87–92%; see Supplementary Fig. S2) and few murine SGCs express Iba-1 (7%; 6–9%).

#### *In vitro* characterization of canine and murine SGCs

**Figure 5:**
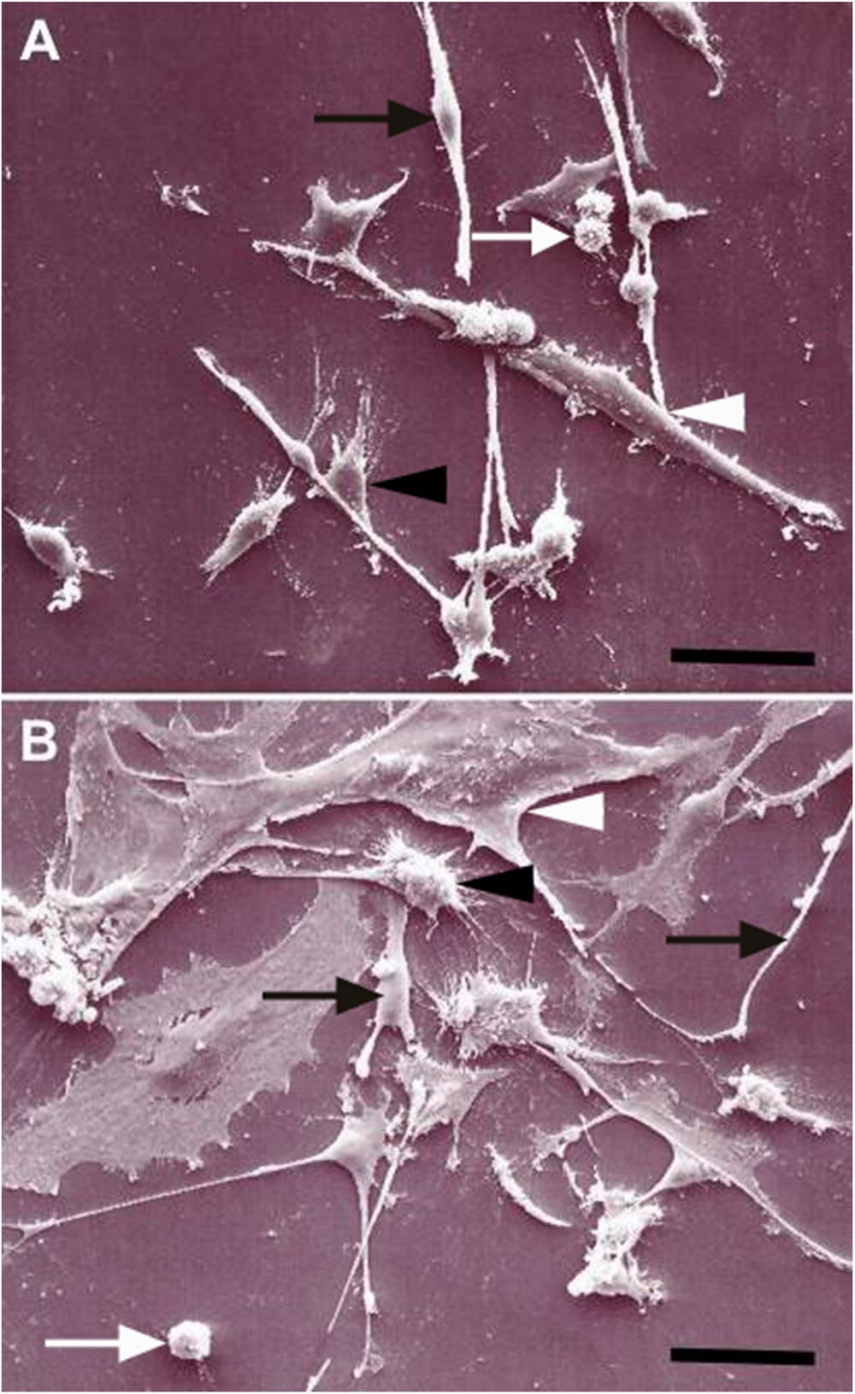
Morphologies of canine (**A**) and murine (**B**) satellite glial cells (SGCs) *in vitro*. Note elongated spindle cells (black arrow), small round cells (white arrow), multipolar cells (black arrowhead), and flattened fibroblastic cells (white arrowhead). Note the large size of fibroblastic cells and low number of round cells in murine compared to canine cultures. Scanning electron microscopy. Bars, 50 µm.

DRG cell cultures contained SGCs, remnants of myelin sheath components and no neurons. Scanning electron microscopy revealed that SGCs of both dogs and mice exhibit morphologically four subtypes including spindeloid, multipolar, flattened fibroblastoid, and small round cells. These subtypes were found in equal numbers in canine cell cultures, whereas murine cell cultures were dominated by equal numbers of spindeloid, multipolar, and fibroblastoid cells. In addition, fibroblastoid cells were considerably larger in murine compared to canine cultures (Fig. 5). Transmission immune-electron microscopy of canine SGCs revealed that the intermediate filament GFAP is predominantly expressed by spindeloid cells (see Supplementary Fig. S4). Immunofluorescence confirmed GFAP expression in a large proportion of canine and murine SGCs and vimentin expression in nearly all canine SGCs (>99%). CNPase was expressed by the vast majority of canine (>84%) and murine (>96%) SGCs. In contrast, beta III tubulin^+^, Iba1^+^, and p75^NTR+^ cells were not detected in canine and murine SGC cultures.

**Figure 6:**
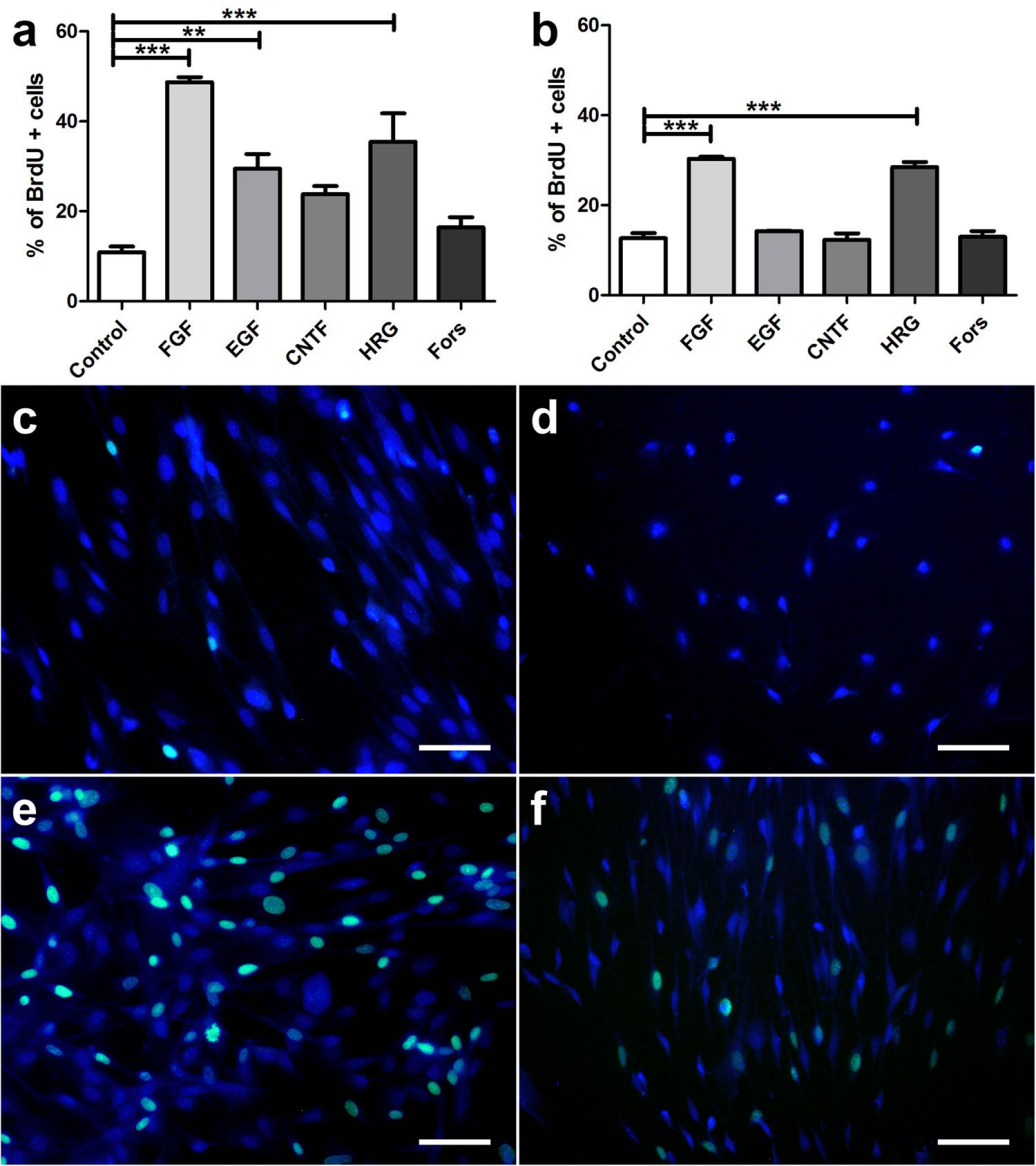
BrdU assay of canine (**a**,**c**,**e**) and murine (**b**,**d**,**f**) satellite glial cells (SGCs) supplemented with fibroblast growth factor 2 (FGF), epidermal growth factor, (EGF), ciliary neurotrophic factor (CNTF), heregulin 1β (HRG), and forskolin (Fors). (**a**,**b**) Graphs show the percentage of BrdU^+^ SGCs with and without supplementation. (**c**–**f**) Immunofluorescence BrdU labelling (green) of SGCs with bisbenzimide as nuclear counterstain. (**c**,**d**) control medium. (**e**,**f**) FGF supplementation. **P < 0.01. ***P < 0.001. Bars, 60 μm. Shown are means with standard errors of the mean (SEM).

**Figure 7:**
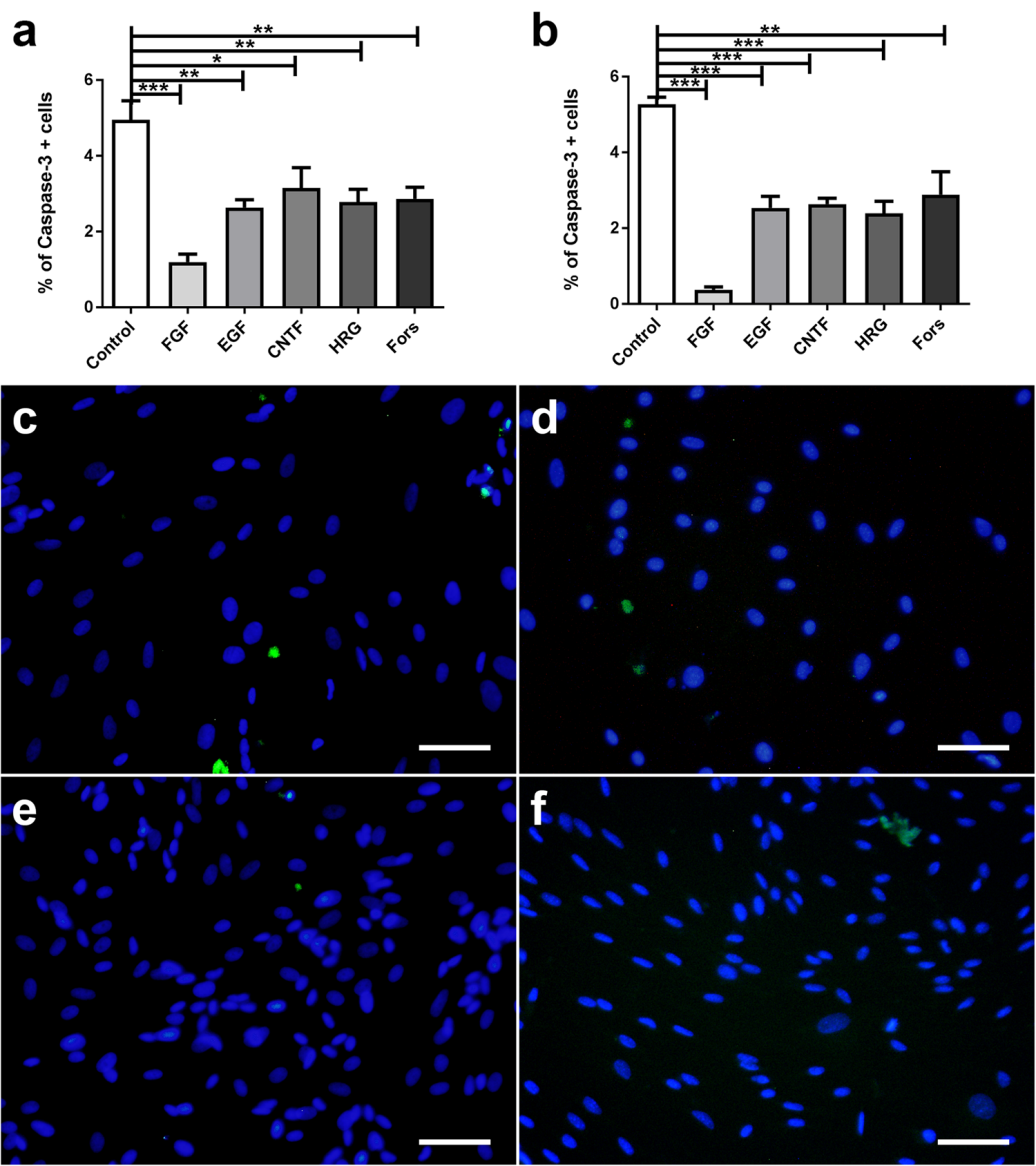
Apoptosis assay of canine (**a**,**c**,**e**) and murine (**b**,**d**,**f**) satellite glial cells (SGCs) supplemented with fibroblast growth factor 2 (FGF), epidermal growth factor, (EGF), ciliary neurotrophic factor (CNTF), heregulin 1β (HRG), and forskolin (Fors). (**a**,**b**) Graphs show the percentage of caspase-3^+^ SGCs with and without supplementation. (**c**–**f**) Immunofluorescence caspase-3 labelling (green) of SGCs with bisbenzimide as nuclear counterstain. (**c**,**d**) control medium. (**e**,**f**) FGF supplementation. *P < 0.05. **P < 0.01. ***P < 0.001. Bars, 60 μm. Shown are means with standard errors of the mean (SEM).

**Figure 8:**
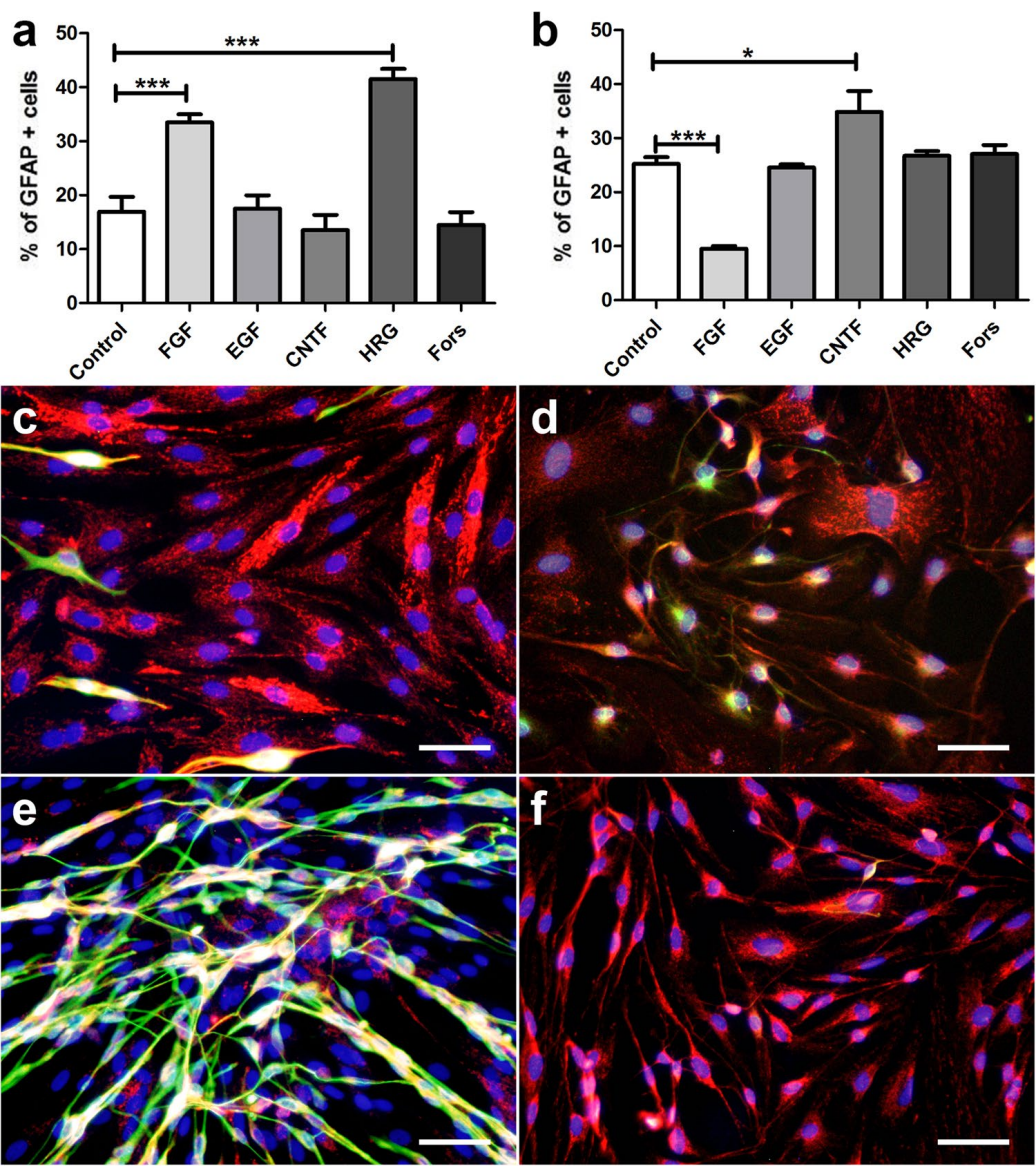
GFAP expression of canine (**a**,**c**,**e**) and murine (**b**,**d**,**f**) satellite glial cells (SGCs) supplemented with fibroblast growth factor 2 (FGF), epidermal growth factor, (EGF), ciliary neurotrophic factor (CNTF), heregulin 1β (HRG), and forskolin (Fors). (**a**,**b**) Graphs show the percentage of glial fibrillary acidic protein (GFAP)^+^ SGCs with and without supplementation. (**c**–**f**) Immunofluorescence double-labelling of SGCs for 2′,3′-cyclic-nucleotide 3′-phosphodiesterase (CNPase; red) and GFAP (green) with bisbenzimide as nuclear counterstain. (**c**,**d**) control medium. (**e**,**f**) FGF supplementation. *P < 0.05. ***P < 0.001. Bars, 60 μm. Shown are means with standard errors of the mean (SEM).

The influence of growth factors and forskolin on canine and murine SGCs with respect to proliferation, degeneration, and differentiation was assessed by evaluation of the number of BrdU^+^, caspase-3^+^, and GFAP^+^ cells, respectively. Canine and murine SGCs showed an increased proliferation rate in the presence of FGF-2 and HRG-1β (Fig. 6). Proliferation of canine in contrast to murine SGCs was also stimulated by EGF supplementation. The number of caspase-3^+^ SGCs was significantly reduced by FGF-2, EGF, HRG-1β, and forskolin supplementation in both species (Fig. 7). FGF-2 had the strongest and CNTF no impact on the number of apoptotic cells. FGF-2 increased or reduced the percentage of GFAP^+^ canine and murine SGCs, respectively (Fig. 8). The percentage of GFAP^+^ SGCs was also increased by HRG-1β supplementation in dogs (Fig. 8a) and CNTF supplementation in mice (Fig. 8b).

**Figure 9:**
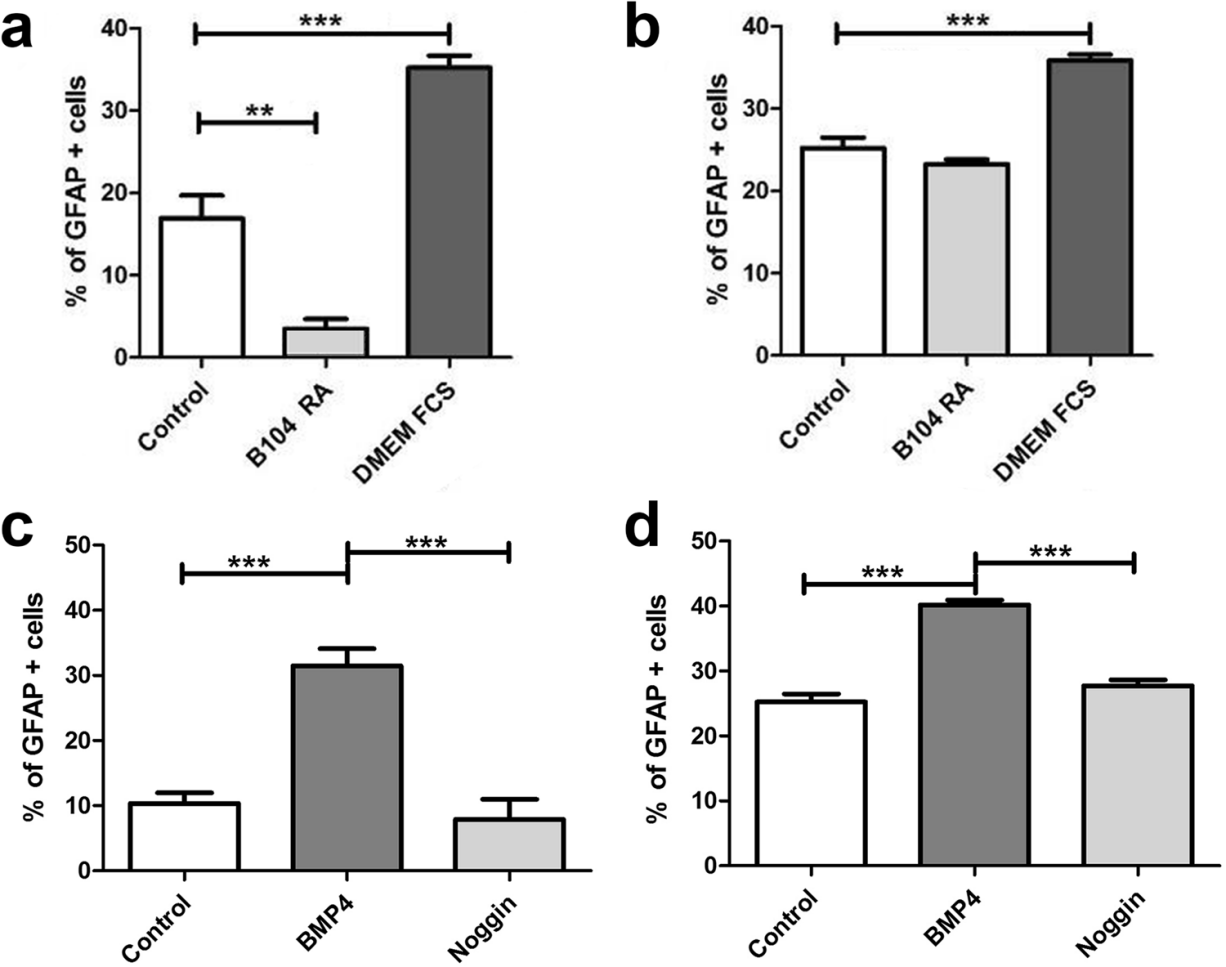
GFAP expression of canine (**a**,**c**) and murine (**b**,**d**) satellite glial cells (SGCs) using different growth media (control, B104 with 5 µM retinoic acid, DMEM with 20% fetal calf serum; **a**,**b**) or supplemented with bone morphogenetic protein 4 (BMP4) and noggin (**c**,**d**). Graphs show the percentage of glial fibrillary acidic protein (GFAP)^+^ SGCs. **P < 0.01. ***P < 0.001. Shown are means with standard errors of the mean (SEM).

To further characterize the influence of external stimuli on SGC differentiation, canine and murine SGCs were incubated with a variety of differentiation media as well as BMP4 and noggin. An astrocytic differentiation medium (DMEM with FCS) increased the percentage GFAP^+^ cells in both species, whereas only canine SGCs showed a significantly reduced percentage of GFAP^+^ cells in an oligodendrocytic differentiation medium (B104-conditioned DMEM with RA) compared to the control medium (Fig. 9a,b). BMP4 increased the percentage of GFAP^+^ cells in both species, whereas no effect was found after noggin supplementation (Fig. 9c,d). CNPase expression was not affected by the investigated culture conditions including growth factor supplementation.

**Figure 10:**
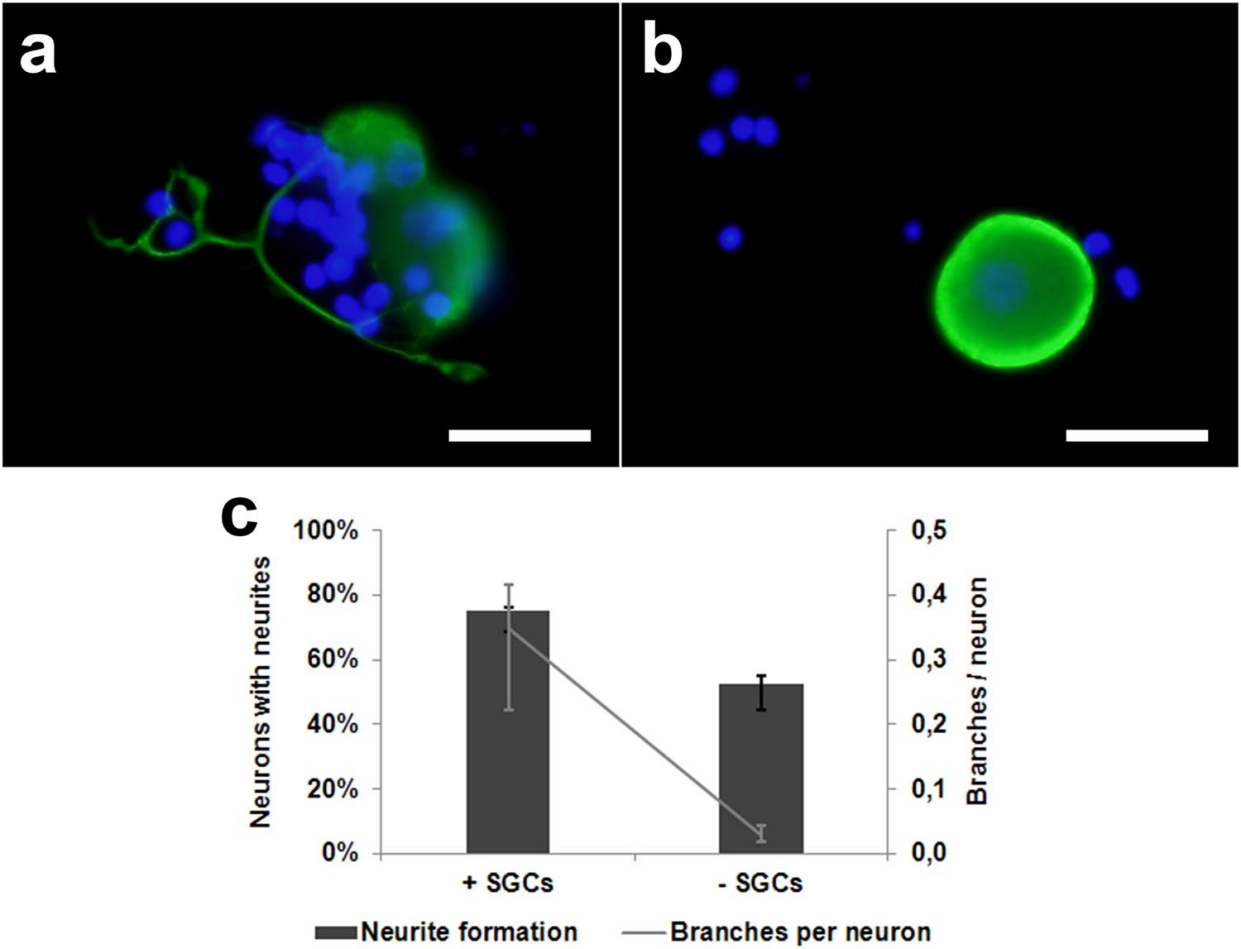
Canine dorsal root ganglion neurons cultured with (**a**) or without (**b**) a satellite glial cell (SGC)-enriched cell fraction at 24 hours post seeding. Immunofluorescence for neuronal class III β-tubulin (green) with bisbenzimide as nuclear counterstain. Note neurite outgrowth under co-culture conditions only. Note the neurite outgrowth in the direction of non-neuronal cells. Bars, 40 μm. (**c**) Percentage of neurons with neurites and number of neurites per neuron in cell culture with or without SGCs. Shown are median values with maximum and minimum.

Interestingly, formation of neuronal processes was found in a significantly higher percentage of canine DRG neurons co-cultured for 24 hours with a SGC-enriched cell fraction compared to conventionally cultured canine neurons (*P* = 0.0002; Fig. 10). Furthermore, arborization of neuronal processes was enhanced by co-culturing (*P* = 0.0003; Fig. 10).

## Discussion

The present study provides the first detailed *in situ* and *in vitro* characterization of canine DRG SGCs. *In situ*, canine SGCs and DRG’s provide a functional unit characterized by intimate intercellular contacts. Though GS has been described as the best marker for SGCs in rats and mice^29^, this is not the case for canine cells. Most canine SGCs were characterized by a strong co-expression of CNPase and GFAP, which represent classical markers of oligodendrocytes and astrocytes, respectively^35^. Interestingly, SGCs of monkeys expressed GS, like murine SGCs, as well as CNPase and GFAP similar to canine SGCs. Canine and simian SGCs also express the intermediate filament vimentin, which is normally found in mesenchymal cells including myelinating and non-myelinating Schwann cells^36^ as well as microglia^5^. A co-expression of GFAP and vimentin seems to be characteristic for less differentiated immature astrocytes in dogs^37^. Similarly, these two markers are expressed by astrocyte precursor cells and immature astrocytes in developing rat retinae^38^. A strong expression of vimentin has also been described in rat SGCs^39–41^. Various studies demonstrated a low expression of CNPase and GFAP in 1- to 2-month-old Sprague Dawley rats, which is up-regulated after nerve damage^42–44^. In contrast, a strong GFAP expression was observed in normal SGCs in a recent study using 3- to 4-month-old Wistar rats^39^. Similarly, a further study found a strong GFAP expression in SGCs of male 8-week-old C57BL/6J mice^45^, whereas no GFAP expression was present in SGCs of 4-month-old C57BL/6NCrl mice^46^. These differences might be caused by the differences in strain and age of the investigated animals or by technical reasons. SGCs of guinea pigs are reported to lack any expression of GFAP^46^. Rat SGCs are also usually positive for S-100 and p75^NTR^, whereas only 11% and 1% of canine SGCs expressed these markers in the present study, respectively^47,48^. S-100 expression of rat SGCs is dependent on size and possibly on the function of the ensheathed neurons^39^.

The present study also demonstrated Iba-1^+^ and CD3^+^ cells in a low percentage of canine SGCs, which might represent immune cells (macrophages and T cells, respectively) in close vicinity to DRG neurons. On the other hand the majority of SGCs in human trigeminal ganglia (>80%) express typical markers of antigen presenting cells including CD40, CD80, CD86, and MHC class II highlighting immune functions of SGCs^33^. MHC class II proteins were also found in 18% of canine SGCs. In addition, these cells showed high expression levels of classical interferon stimulated genes including ISG15, PKR, and OAS1, which act as major antiviral effector proteins^49,50^. Consequently, SGCs seem to be involved in the innate and adaptive immune reactions in order to protect the ensheathed neurons against invading pathogens especially viruses.

A highly interesting feature of canine SGCs was their expression of the intermediate filament nestin and the transcription factor Sox2. Nestin is used as a marker of neural stem/progenitor cells and can be found in various CNS cells during the embryonic period of ontogenesis^51^. In adult animals, nestin-immunopositive cells have been found in so-called germinative zones of the brain such as the subventricular zone of the lateral ventricles and the dentate gyrus of the hippocampus as well as under pathological conditions^51^. Sox2 is expressed in neurogenic regions of the CNS and maintains the neural stem cell state by controlling proliferation and differentiation^52,53^. In the developing PNS, Sox2 regulates the migration, proliferation, and differentiation of neural crest stem cells, which give rise to Schwann cells, DRG neurons, and SGCs^54,55^. In the adult PNS, Sox2 seems to play a cell-type specific role, which is mediated by different down-stream targets of this transcription factor^56^. A nuclear Sox2 expression was also described in non-myelinating Schwann cells as well as neural progenitor cells and normal or reactive astrocytes of adult rats^57^. Interestingly, previous studies demonstrated that adult rat SGCs strongly express Sox2 and have the potential to generate neurospheres^58^. Moreover, SGCs isolated from embryonic and neonatal rat DRGs have the capacity to transform into astrocytes, oligodendrocytes, and Schwann cells^59^. All these findings underline the chimeric character and high plasticity of SGCs unifying properties of central and peripheral glia cell populations. The plasticity of canine SGCs was confirmed *in vitro*, where isolated cells developed a variety of morphologic features. Nearly all canine SGCs showed strong CNPase expression, whereas predominantly spindeloid cells expressed GFAP. No Iba-1^+^ or p75^NTR+^ cells were detected in the present canine and murine DRG cell cultures demonstrating the absence of macrophages and Schwann cells, respectively^8,23^. Nevertheless, the cultures might contain fibroblasts, which form an integral part of the DRG connective tissue and are also found in canine mixed brain cell cultures^60^.

The morphologic features of murine SGC cultures generally resembled their canine counterpart but the former differed by larger size of fibroblastoid cells and lower number of round cells. These differences stimulated additional *in vitro* experiments to characterize and compare phenotypical features canine and murine SGCs. The percentage of GFAP^+^ canine and murine SGCs was strongly influenced by the respective culture conditions, whereas high CNPase expression was unaffected. An astrocytic differentiation medium stimulated GFAP expression in SGCs of both species as expected. Nevertheless, an oligodendrocytic differentiation medium decreased the percentage of GFAP^+^ canine but not murine SGCs. GFAP expression was also induced by CNTF in murine and HRG-1β in canine SGCs. Moreover, GFAP expression was reduced by FGF-2 in murine but induced in canine SGCs. These results demonstrate species-specific differences in the reaction pattern of SGCs to external stimuli such as growth factors. The proliferation of canine and murine SGCs was induced by FGF-2 and HRG-1β, which both activate mitogen-activated protein kinase (MAPK) signaling pathways and induce the proliferation of canine and rodent Schwann cells and OECs^23,61^. Interestingly, canine SGCs also reacted to supplementation with EGF, which stimulates proliferation, survival, migration and differentiation into the oligodendrocyte lineage^62^. However, GFAP expression was induced in SGCs of both species by BMP4, which is a member of the transforming growth factor (TGF)-β family and known for his ability to drive the differentiation of neural stem cells towards an astrocytic fate^63^. Consequently, BMP4 cellular signaling pathways seem to be similar in canine and murine SGCs and influence astrocytic differentiation of these cells.

Finally, the present study demonstrated that SGCs of adult dogs support neurite outgrowth of DRG neurons *in vit*ro, whereas a previous study reported dendritic outgrowth from neonatal or juvenile rat nodose ganglion neurons only in the absence of SGCs^64^. This growth promoting effect observed in dogs could be a species-specific property of canine SGCs mediated by the production and release of NGF and/or other neurotrophins. Consequently, canine SGCs might represent a promising candidate for cell transplantation studies of degenerative CNS diseases. However, the age of the investigated animals might also cause differences in the effects of SGCs on neurons. Furthermore, SGCs may mask or inhibit neuronal growth factor receptors *in vivo*, a condition lost in dissociated DRG cultures^29^. Interestingly, an axotomy associated sympatho-sensory sprouting is observed in sensory ganglia, which is discussed in the pathogenesis of sympathetically maintained chronic pain^65^. If the underlying neurotrophic signals originate from neurons and/or SGCs is a current focus in pain research.

In conclusion, the current study revealed the presence of a CNPase, GFAP, vimentin, nestin, and Sox2 co-expressing glial cell population in DRGs of adult dogs. This canine SGC population warrants further investigation, especially with regard to the differentiation potential and neurotrophic properties of these cells and the existence of an analogous cell population in monkeys and humans^33^. The easy accessibility and the phenotypic characteristics highlight this glial population as a promising candidate for novel therapeutic concepts, such as cell transplantation techniques.

## Methods

### Animals

Antigen-specific immunoreaction of primary canine satellite glial cells was evaluated in DRGs of five healthy adult Beagle dogs (from each dog one DRG of the 3^rd^ cervical nerve, Table 1 dogs no. 1–5). Electron microscopy was performed in a DRG of the 3^rd^ cervical nerve of one healthy adult Beagle dog (Table 1 dog no. 6). DRGs of cervical, thoracic, and lumbar spinal cord segments from four healthy adult Beagle dogs (Table 1 dogs no. 7–10) were collected at postmortem examination for primary DRG cell culture. Furthermore, electron microscopy was performed (Table 1 dog no. 10). In addition, primary cells of four other healthy adult Beagle dogs were used for DRG co-culture experiments (Table 1 dogs no. 11–14). All previously performed studies in dogs were unrelated to the present project. The samples were taken post mortally.

**Table 1:** Details of the dogs investigated in the different experiments.

Dog No.	Breed	Age	Sex	Experiment
1	Beagle	1 year	male	*In situ*
2	Beagle	1 year	female	*In situ*
3	Beagle	1 year	female	*In situ*
4	Beagle	1 year	female	*In situ*
5	Beagle	1 year	male	*In situ*
6	Beagle	1 year	male	*In situ*
7	Beagle	6 months	male	*In vitro*
8	Beagle	6 months	female	*In vitro*
9	Beagle	23 months	male	*In vitro*
10	Beagle	22 months	female	*In vitro*
11	Beagle	8–9 months	male	Co-culture
12	Beagle	8–9 months	female	Co-culture
13	Beagle	8–9 months	male	Co-culture
14	Beagle	8–9 months	male	Co-culture

The experiments were in compliance with the law of animal welfare of Lower-Saxony and North Rhine-Westphalia, Germany and approved by Lower Saxony State Office for Consumer Protection and Food Safety, permission numbers: 33.9-42502-05-12A241; 33.9-42502-05-14A443; and Ministry for Climate Protection, Environment, Agriculture, Conservation and Consumer Protection of the State of North Rhine-Westphalia, permission number: A0164/91. The animals did not suffer from diseases affecting the nervous system as determined by clinical examination.

For the murine studies nine male 4-month-old C57BL/6NCrl mice (AnLab s.r.o, Prague, Czech Republic) were euthanized according to the law of animal welfare of Lower Saxony (permission number: 42500/1H). Dorsal root ganglia were collected for paraffin-embedding (5 mice) and isolation of primary murine satellite cell (4 mice).

Furthermore, archived DRGs of three adult non-human primates (1 *Cebus capucinus*, 1 *Macaca nemestrina*, 1 *Semnopithecus entellus)* from the archive of routine necropsy cases of the department of pathology, which died due to spontaneous unrelated diseases, were immunohistologically investigated. Samples for histology and immunohistochemistry were fixed in 4% formaldehyde, routinely embedded in paraffin^66^. 3–5 µm sections were routinely stained with hematoxylin and eosin or mounted on SuperFrost^®^ glass slides for immunohistochemistry. No cells were obtained from non-human primates. All animal procedures were performed in accordance with the German regulations and legal requirements.

### Immunohistochemistry

Immunohistochemistry was performed as described^67,68^. Briefly, formalin-fixed, paraffin-embedded tissue sections were treated with 0.5% H_2_O_2_ to block endogenous peroxidase, depending on the antibody heated in sodium citrate buffer, and incubated with 20% goat serum to block non-specific binding sites. Subsequently, sections were incubated with the respective primary antibody (Ab, Table 2) overnight at 4 °C. Negative control sections were incubated with rabbit serum (R4505; Sigma-Aldrich, Taufkirchen, Germany) or mouse IgG1 isotype control (CBL600; Millipore, Schwalbach, Germany). Biotinylated goat-anti-rabbit IgG (BA-1000) or goat-anti-mouse IgG (BA-9200) diluted 1:200 (Vector Laboratories, Burlingame, CA, USA) were used as secondary antibodies (Abs). After visualization of the antigen-antibody reaction using the avidin-biotin-peroxidase complex (ABC) method (Vector Laboratories) and the chromogen 3,3′-diamino-benzidine (DAB) sections were slightly counterstained with Mayer’s hematoxylin.

**Table 2:** Summary of primary antibodies used for the phenotyping of satellite glial cells.

Antibody	Primary antibody	Species investigated	Pretreatment (IHC) and dilution (IHC/IFC)
βIII tubulin	Covance Inc., MRB-435P, rAb	dog	−/1:1000
Cleaved caspase-3	Cell Signaling, 9661, pAb	dog, mouse	−/1:500
CNPase	Millipore, MAB326, mAb	dog, mouse, NHP	Citrate buffer/microwave 1:100/1:500
GFAP	Dako, Z0334, pAb	dog, mouse, NHP	No pretreatment 1:2000/1:400
Glutamine synthetase	Santa Cruz, sc-9067, pAb	dog, mouse, NHP	Citrate buffer/microwave 1:100/-
Vimentin	Dako, M0725, mAb	dog, NHP	No pretreatment 1:100/-
Nestin	Acris, 1484-1500, pAb	dog, mouse	Citrate buffer/microwave 1:500/-
Sox2	Cell Signaling, 3579, rAb	dog	Citrate buffer/microwave 1:50/-
HNK-1	Sigma-Aldrich, C6680, mAb	dog	No pretreatment 1:500/-
p75^NTR^	ATCC, Clone HB8737, mAb	dog	No pretreatment 1:5/-
Periaxin	Sigma-Aldrich, HPA001868, pAb	dog	Citrate buffer/microwave 1:5000/-
S-100	Sigma-Aldrich, S2644, pAb	dog	No pretreatment 1:800/-
CD3	Dako, A0452, pAb	dog	Citrate buffer/microwave 1:2000/-
CD79α	Dako, M7051, mAb	dog	Citrate buffer/microwave 1:60/-
Iba-1	Wako Chemicals, 019-19741, pAb	dog	Citrate buffer/microwave 1:100/-
MHC II	Dako; TAL1B5,mAb	dog	Citrate buffer/microwave 1:100/-
Pax5	BD Biosciences, 610865, mAb	dog	Citrate buffer/microwave 1:200/-
ISG15	Santa Cruz, sc-50366, pAb	dog	Citrate buffer/microwave 1:600/-
Mx	Georg Kochs, M143, mAb	dog	Citrate buffer/microwave 1:1000/-
OAS1	Santa Cruz, sc-98424, pAb	dog	Citrate buffer/microwave 1:600/-
PKR	Abcam, ab32036, rAb	dog	Citrate buffer/microwave 1:600/-
Stat1 (p84/p91)	Santa Cruz, sc-346, pAb	dog	No pretreatment 1:400/-
Stat2	Santa Cruz, sc1668, mAb	dog	Citrate buffer/microwave 1:100/-

CNPase, 2′,3′-cyclic-nucleotide 3′-phosphodiesterase; GFAP, glial fibrillary acidic protein; HNK-1, human natural killer-1; ISG15, interferon stimulated gene 15; MHC, major histocompatibility complex; OAS1, 2′-5′ oligoadenylate synthetase 1; PKR, protein kinase R; Stat, signal transducer and activator of transcription; IFC, immunofluorescence; IHC, immunohistochemistry; NHP, non-human primate; mAb, mouse monoclonal antibody; pAb, rabbit polyclonal antibody; p75^NTR^, low affinity neurotrophin receptor; rAb, rabbit monoclonal antibody

**Table 3:** Comparative analysis of the *in situ* expression of CNPase, GFAP, and glutamine synthetase in canine, murine, and simian satellite glial cells. Shown are the median percentages of positive cells with range.

Marker	Canine SGCs	Murine SGCs	Simian SGCs
CNPase	**93%** (86–97%)	**5%** (4–6%)	**92%** (85–94%)
GFAP	**78%** (73–89%)	**0%** (0–0%)	**80%** (78–84%)
Glutamine Synthetase	**44%** (25–52%)	**71%** (70–72%)	**94%** (90–98%)

CNPase = 2′,3′-cyclic-nucleotide 3′-phosphodiesterase; GFAP = glial fibrillary acidic protein; SGCs = satellite glial cells.

The percentage of immunopositive SGCs was determined by counting the number of immunopositive and immunonegative SGCs adjacent to at least 10 randomly selected neurons in each investigated canine, murine, and simian DRG.

For immunofluorescence on paraffin-embedded DRGs pretreatment and blocking procedure were performed as previously described^15^. The slides were incubated for 1 hour at room temperature with the respective primary antibodies (Table 2). After one washing step the slides were incubated with the secondary antibodies, respectively (Goat-anti-mouse Cy3, 1:200; Goat-anti-rabbit Cy2, 1:200; Jackson Immunoresearch, Dianova, Hamburg, Germany). Nuclei were counterstained with 0.01% bisbenzimide Hoechst 33258 (Sigma-Aldrich) for 10 min at room temperature.

### Isolation of canine and murine satellite glial cells

For the isolation of SGCs 30–40 DRGs from each dog or 20–30 DRGs from each mouse were collected and stored in cold phosphate buffered saline (PBS), the fibrous capsule and blood vessels were removed and ganglia were dissected into small pieces. Afterwards the ganglia were incubated for enzymatic dissociation for 30 min at 37 °C with IV-S hyaluronidase (H3884), type IV collagenase (C5138) and type XI collagenase (C7657; Sigma-Aldrich) in a 0.2% solution (each enzyme) in 1x Hank’s balanced salt solution (HBSS; Gibco^®^, Invitrogen, Darmstadt, Germany). After 30 min, type I trypsin (T8003) was added (0.2% solution) followed by 30 min incubation at 37 °C. Subsequently, the tissue was mechanically dissociated by adding DNase I (0.2%; Sigma-Aldrich) using successively narrowed flame-polished Pasteur pipettes, pelleted by centrifugation (5 min, 300 × g, 4 °C), and re-suspended in Dulbecco’s modified eagle medium (DMEM; Gibco^®^, Invitrogen) with 10% fetal calf serum (FCS; Biochrom AG, Berlin, Germany) and 1% penicillin-streptomycin (PS; PAA Laboratories GmbH, Pasching, Austria). Cells were seeded at a density of 10.000 cells/well in 96 ½ titer plates (Nunclon, Thermofisher, Schwerte, Germany) coated with poly-L-lysine (PLL, 0.1 mg/ml, Sigma-Aldrich).

To investigate the functional aspect of the SGCs with respect to neurite outgrowth, canine DRG neurons were isolated^66^ and co-cultured with an SGC-enriched fraction (Suppl. Fig. 1). Neurons were stained with beta III-tubulin and the number of processes formed was counted^69^.

### Transmission and scanning electron microscopy

For transmission electron microscopy, complete DRGs or isolated cells were fixed for 24 h in 2.5% glutaraldehyde solution, rinsed with 0.1% sodium cacodylate buffer (pH 7.2), postfixed in 1% osmium tetroxide, and embedded in EPON 812 (Serva, Heidelberg, Germany) as described^25,70^.

For transmission immune-electron microscopy of SGCs, cells were initially fixed with 4% paraformaldehyde with 0.25% glutaraldehyde in 1% sodium cacodylate buffer, treated with 0.05 M glycine and 0.25% Triton X-100, blocked with 5% goat serum, incubated with an polyclonal rabbit antibody directed against GFAP (Z0334; Dako, Hamburg, Germany) at 4 °C overnight and sequentially incubated with a gold particles-labeled goat anti-rabbit secondary antibody for 1 hour at room temperature. Subsequent fixation and embedding were performed as described^25,70^. Sections and SGCs were stained with lead citrate and uranyl acetate and investigated using an EM 10c (Carl Zeiss Jena GmbH, Oberkochen, Germany).

For scanning electron microscopy, isolated SGCs were cultured on glass slides coated with PLL. The cells were fixed in 4% paraformaldehyde with 0.25% glutaraldehyde in 1% sodium cacodylate buffer. Afterwards the samples were dehydrated in a series of graded ethanol, dried and coated in a sputter-coater (SCD 040; Oerlikon Balzers, Balzers, Liechtenstein) with gold. For visualization a digital scanning microscope (DSM 940, Carl Zeiss Jena GmbH) was used.

### Immunocytochemistry

Cells were fixed in 4% paraformaldehyde and permeability was achieved by incubation in PBS plus 0.25% Triton X-100. Afterwards cells were incubated with the respective primary antibody (Table 2) for 2 hours at room temperature. Followed after a short washing step by the incubation with the secondary antibody. Cy3-labeled goat-anti mouse (1:200; Jackson Immunoresearch) was used for monoclonal mouse antibodies and Cy2-labeled goat-anti rabbit (1:200; Jackson Immunoresearch) was used for polyclonal rabbit antibodies, respectively. Nuclei were counterstained with 0.01% bisbenzimide Hoechst 33258 (Sigma-Aldrich) for 5 min at room temperature.

### Cell proliferation assays

The proliferation rate of SGCs (passage 2) supplemented with fibroblast growth factor 2 (FGF-2, 40 ng/ml; recombinant human, Peprotech, Hamburg, Germany), epidermal growth factor (EGF, 40 ng/ml; R&D Systems, Wiesbaden-Nordenstadt, Germany), ciliary neurotrophic factor (CNTF; 40 ng/ml; Peprotec), heregulin 1β (HRG-1β, 40 ng/ml; R&D Systems), and the cAMP-elevator forskolin (4 µM; F6886; Sigma-Aldrich) was evaluated. SGCs were seeded at a density of 2.500 cells/well in a 96 1/2 well titer plate coated with PLL. Cultures were maintained under standard condition for 60 h with the respective growth factor and BrdU (100 μM; 5-Bromo-2′-deoxy-uridine labelling and detection kit III, Sigma-Aldrich) was applied for 12 h prior to fixation. Subsequently, cells were fixed and immunostained according to the manufacturer’s protocol. Proliferating cells were stained with Cy2-coupled goat anti-rabbit antibodies (1:100; Jackson Immunoresearch,) and nuclei were counterstained with 0.01% bisbenzimide Hoechst 33258 (Sigma-Aldrich) for 5 min at room temperature. Triplicate experiments were performed and four high-power field photos from an inverted fluorescence microscope (Olympus IX-70, Hamburg, Germany) per well were manually counted with Image J (National Institutes of Health, Bethesda, MD, USA). The percentage of BrdU positive cells was compared to the total number of cells counted. Additionally, the number of GFAP and CNPase positive cells was evaluated.

### Differentiation assay

SGCs (2,500 cells/well, passage 2) were seeded on PLL coated ½ wells in a 96 well plate and cultured in DMEM containing 10% FCS (control), B104-conditioned DMEM containing 5 μM all-trans retinoic acid (RA; oligodendrocytic differentiation medium), or DMEM containing 20% FCS (astrocytic differentiation medium), respectively for three days^71^.

Furthermore, the effect of bone morphogenetic protein 4 (BMP4) and its inhibitor noggin was investigated. Analogue to the previously described experiment SGCs (passage 2) were cultivated and supplemented with BMP4 (10 ng/ml, R&D Systems,) or the BMP4 inhibitor noggin (250 ng/ml; R&D Systems). The experiments were performed in triplicates and number of GFAP positive cells was evaluated.

### Statistical analysis

Statistical analysis was performed using GraphPad software (Prism 5; GraphPad Software, Inc., La Jolla, CA, USA). Data were analyzed for multiple comparisons by a one-way ANOVA followed by Bonferroni’s multiple comparisons test. Results of the co-culture experiment were analyzed by Student’s t tests for paired samples. *P* values < 0.05 were considered statistically significant.

## Electronic supplementary material

Supplementary Information
