# Supplementary material for: Canine dorsal root ganglia satellite glial cells represent an exceptional cell population with astrocytic and oligodendrocytic properties
Source: Sci Rep. 2017 Oct 24;7:13915. doi: 10.1038/s41598-017-14246-7 (PMC5654978; doi:10.1038/s41598-017-14246-7)

Supplementary Information

Canine dorsal root ganglia satellite glial cells represent an exceptional cell population with astrocytic and oligodendrocytic properties

W. Tongtako, A. Lehmbecker, Y. Wang, K. Hahn, W. Baumgärtner, I. Gerhauser

Supplementary Fig. S1: Preparation of a pooled satellite glial cell (SGC)-enriched cell fraction of 4 dogs. Isolated dorsal root ganglia from one dog were splitted on 2 tubes for digestion and subsequent 25% and 27% Percoll centrifugation. About 4 ml of the myelin-poor fraction were collected per tube (in total approximately 16 ml for each dog). For 1:3 dilution, the 16 ml were pipetted in a 50 ml Falcon tube and filled with Dulbecco's modified eagle medium (DMEM) with 1% Penicillin/Streptomycin (PS) and 10% fetal calf serum (FCS) to a volume of 48 ml (4 tubes for 4 dogs). The following centrifugation (5 min at 300 x g and 4°C) leads to a cell pellet containing SGCs, other non-neuronal cells, and small to moderate numbers of neurons. The cell pellet of one dog was resuspended in 2 ml DMEM with 1% PS and 10% FCS. This suspension was used to resuspend consecutively cell pellets of the other dogs and finally contains the non-neuronal cells of four dogs in 2 ml DMEM with 1% PS and 10% FCS. Two tubes containing 5 ml 25% Percoll were prepared, layered with 1 ml of the SGC-enriched resuspension, and centrifuged (15 min at 450 x g and 4°C). The resulting cell pellet contains the remaining neurons. About 4.5 ml of the supernatant were collected from the two tubes, pooled in a 50 ml Falcon tube, and diluted 1:3 by adding 18 ml DMEM with 1% PS and 10% FCS. The following centrifugation (5 min at 300 x g and 4°C) results in a cell pellet containing SGC and other non-neuronal cells, which was resuspended in Sato´s Medium with 0.25% bovine serum albumin (BSA). The cell content was evaluated using a Neubauer chamber.

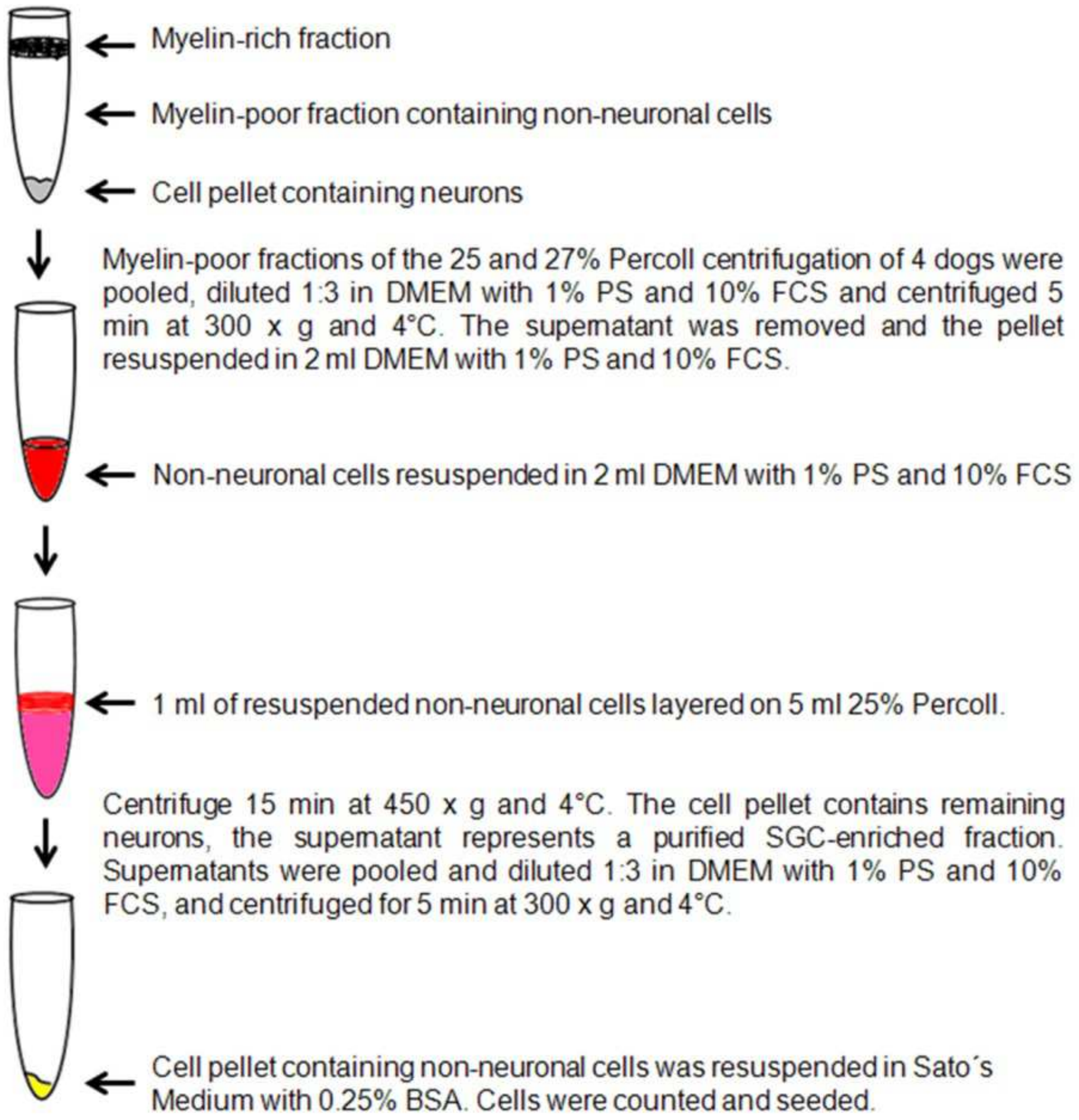
Myelin-rich fraction
Myelin-poor fraction containing non-neuronal cells
Cell pellet containing neurons
Myelin-poor fractions of the 25 and 27% Percoll centrifugation of 4 dogs were pooled, diluted 1:3 in DMEM with 1% PS and 10% FCS and centrifuged 5 min at 300 x g and 4°C. The supernatant was removed and the pellet resuspended in 2 ml DMEM with 1% PS and 10% FCS.
Non-neuronal cells resuspended in 2 ml DMEM with 1% PS and 10% FCS
1 ml of resuspended non-neuronal cells layered on 5 ml 25% Percoll.
Centrifuge 15 min at 450 x g and 4°C. The cell pellet contains remaining neurons, the supernatant represents a purified SGC-enriched fraction. Supernatants were pooled and diluted 1:3 in DMEM with 1% PS and 10% FCS, and centrifuged for 5 min at 300 x g and 4°C.
Cell pellet containing non-neuronal cells was resuspended in Sato´s Medium with 0.25% BSA. Cells were counted and seeded.

Supplementary Fig. S2:

Dorsal root ganglion of a 1-year-old beagle dog **(a, c, d)** and a 4-year-old northern plains gray langur (*Semnopithecus entellus*) **(b)**. The majority of canine **(a)** and simian **(b)** satellite glial cells (SGCs) expressed vimentin. Few canine SGCs also expressed S-100 **(c)**. Most of canine SGCs showed a strong intranuclear expression of Sox2 **(d)**. Bars, 40 µm.

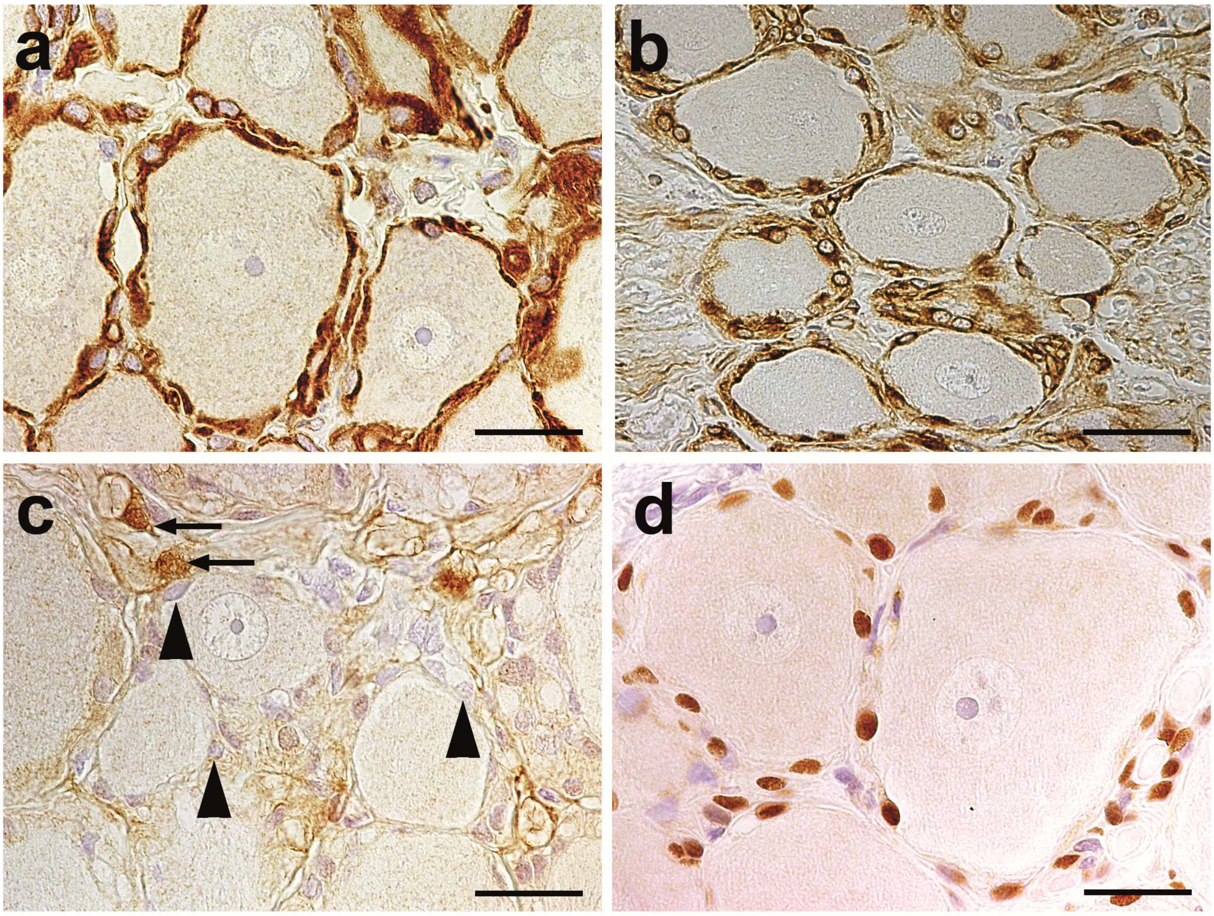

Supplementary Fig. S3:

Dorsal root ganglion of a 4-month-old mouse **(a)** and a 4-year-old northern plains gray langur (*Semnopithecus entellus*) **(b)**. Multiple neurons surrounded by a satellite glial cell sheath. Note few fibroblasts and capillaries in the interstitial stroma. Hematoxylin and eosin staining. Bars, 40 µm.

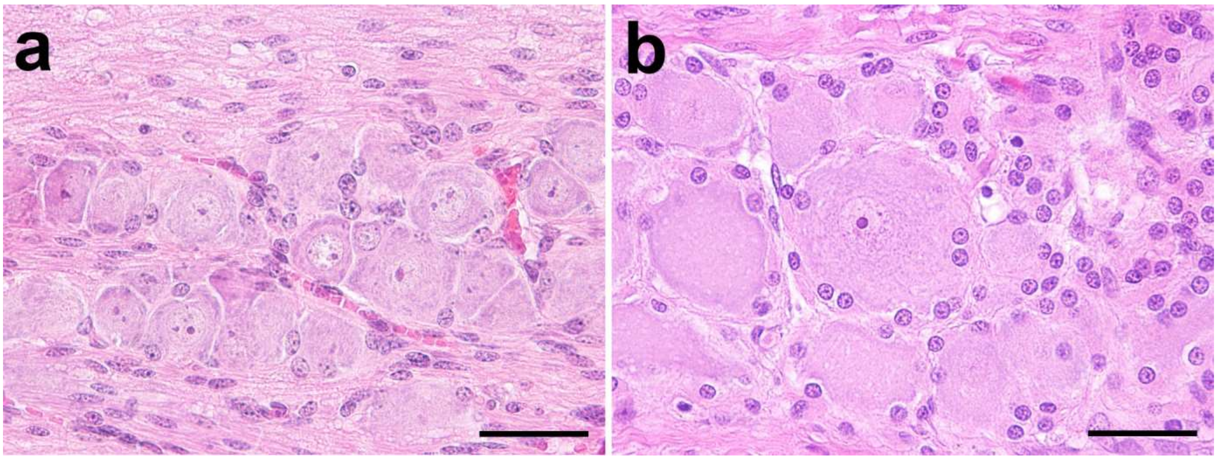

Supplementary Fig. S4:

Dorsal root ganglion of a female 22-month-old beagle dog. Immune transmission electron microscopy for the intermediate filament glial fibrillary acidic protein (GFAP). **(a)** Morphology of GFAP^{+} satellite glial cells *in vitro*. The marked area is shown in a higher magnification in "**b**". Bar, 1 µm. **(b)** Higher magnification of the marked area in "**a**" showing the cytoplasmic GFAP filaments labeled with gold particles (arrows). Bar, 200 nm.

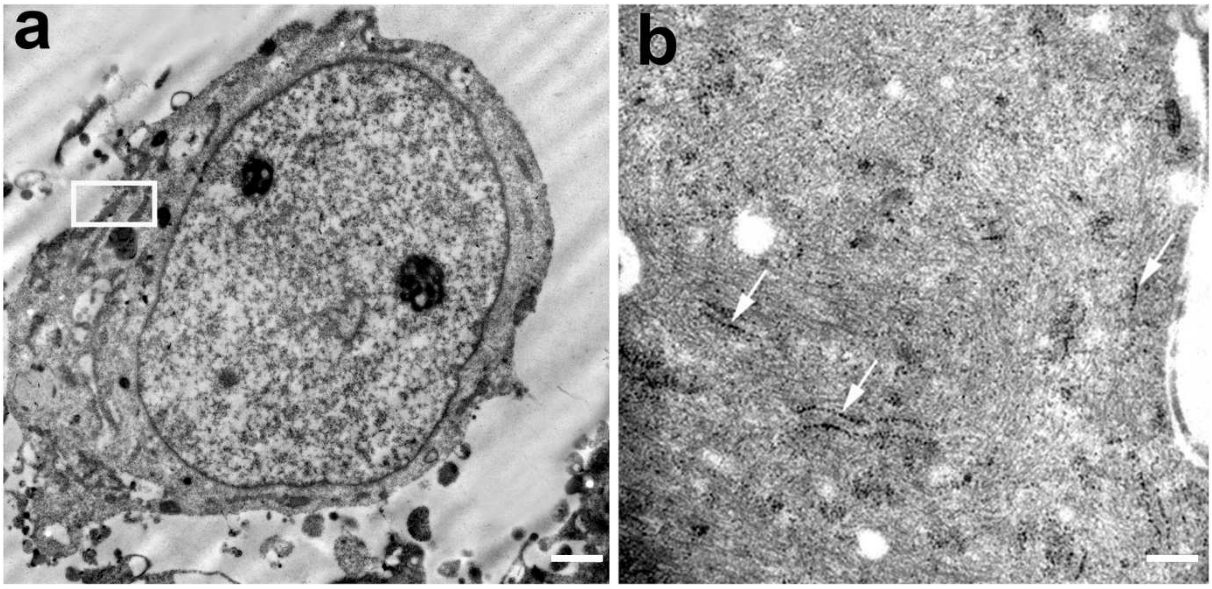

Supplement: Supplementary file 1 — Supplementary Information [file 41598_2017_14246_MOESM1_ESM.pdf]
